# A Comparative Study of Machine Learning Algorithms in Predicting Severe Complications after Bariatric Surgery

**DOI:** 10.3390/jcm8050668

**Published:** 2019-05-12

**Authors:** Yang Cao, Xin Fang, Johan Ottosson, Erik Näslund, Erik Stenberg

**Affiliations:** 1 Clinical Epidemiology and Biostatistics, School of Medical Sciences, Örebro University, 70182 Örebro, Sweden; 2 Unit of Biostatistics, Institute of Environmental Medicine, Karolinska Institutet, 17177 Stockholm, Sweden; fx63783985@hotmail.com; 3 Department of Surgery, Faculty of Medicine and Health, Örebro University, 70182 Örebro, Sweden; johan.ottosson@regionorebrolan.se (J.O.); erik.stenberg@regionorebrolan.se (E.S.); 4 Division of Surgery, Department of Clinical Sciences, Danderyd Hospital, Karolinska Institutet, 18288 Stockholm, Sweden; erik.naslund@ki.se

**Keywords:** machine learning, bariatric surgery, severe complication, prediction, comparative study

## Abstract

Background: Severe obesity is a global public health threat of growing proportions. Accurate models to predict severe postoperative complications could be of value in the preoperative assessment of potential candidates for bariatric surgery. So far, traditional statistical methods have failed to produce high accuracy. We aimed to find a useful machine learning (ML) algorithm to predict the risk for severe complication after bariatric surgery. Methods: We trained and compared 29 supervised ML algorithms using information from 37,811 patients that operated with a bariatric surgical procedure between 2010 and 2014 in Sweden. The algorithms were then tested on 6250 patients operated in 2015. We performed the synthetic minority oversampling technique tackling the issue that only 3% of patients experienced severe complications. Results: Most of the ML algorithms showed high accuracy (>90%) and specificity (>90%) in both the training and test data. However, none of the algorithms achieved an acceptable sensitivity in the test data. We also tried to tune the hyperparameters of the algorithms to maximize sensitivity, but did not yet identify one with a high enough sensitivity that can be used in clinical praxis in bariatric surgery. However, a minor, but perceptible, improvement in deep neural network (NN) ML was found. Conclusion: In predicting the severe postoperative complication among the bariatric surgery patients, ensemble algorithms outperform base algorithms. When compared to other ML algorithms, deep NN has the potential to improve the accuracy and it deserves further investigation. The oversampling technique should be considered in the context of imbalanced data where the number of the interested outcome is relatively small.

## 1. Introduction

Severe obesity is a global public health threat of growing proportions [1]. Bariatric surgery offers the best chance for long-term weight-loss and the resolution of comorbidities [2]. Although modern bariatric surgery is considered to be safe, severe postoperative complications still occur [3,4]. Accurate prediction models for severe postoperative complications could aid in preoperative decision making for surgeons, anesthesiologists, and patients. These models could also serve as the basis for case-mix comparisons between different centers. Some prediction models that are based on the linear regression of patient-specific data allow for relatively simple and interpretable inference; however, so far they have been proven inaccurate and thus cannot be used in clinical practice [5,6].

In contrast, some machine learning (ML) methods have been shown to provide quite accurate predictions and have increasingly been used in the diagnosis and prognosis of different diseases and health conditions [7,8,9]. ML methods are data-driven analytic approaches that specialize in the integration of multiple risk factors into a predictive algorithm [10]. Over the past several decades, ML tools have become more and more popular for medical researchers. A variety of ML algorithms, including artificial neural networks [11], decision trees [12], Bayesian networks [13], and support vector machines (SVMs) [14], have been widely applied with the aim of detecting key features of the patient conditions and modeling the disease progression after treatment from complex health information and medical datasets. The application of different ML methods in feature selection and classification in multidimensional heterogeneous data can provide promising tools for inference in medical practices [15,16]. These highly nonlinear approaches have been utilized in medical research for the development of predictive models, resulting in effective and accurate decision making [17,18,19].

Although new and improved software packages have significantly eased the implementation burden for many ML methods in recent years, few studies have used ML methods to examine the risk factors or predict the prognosis after bariatric surgery, including diabetes remission [20,21], complication [22], weight status [23,24], and adverse events and death [25]. Even though there is evidence that the use of ML methods can improve our understanding of postoperative progression of bariatric surgery, further validation is needed in order for these methods to be considered in clinical practice.

Our study was based on the data from the Scandinavian Obesity Surgery Registry (SOReg). The SOReg is a national quality and research register, which covers virtually all bariatric surgical procedures that have been performed in Sweden since 2010. The register has been described in detail elsewhere [3,26], and a prediction model that is based on logistic regression for the same group of patients has been described previously [6]. As the prediction based on the logistic regression showed very low sensitivity <5% [6], the aim of the current study was to find an algorithm or algorithms that perform well in terms of both sensitivity and specificity, not only on the training data, but also on the test data that were not used to train the algorithms. We applied and compared an array of existing standard supervised ML methods for the unique scientific problem in modeling severe postoperative complication after bariatric surgery.

No human subjects were involved in our study. The study only used deidentified historical registry data for statistical analysis and machine learning, and no member of our research team named in the author list of the paper had access to identifying patient information when analysing the data. The Regional Ethics Committee in Stockholm approved the use of historical registry data (approval number: 2013/535-31/5).

## 2. Materials and Methods

### 2.1. Patients and Features

Patients that were registered in the SOReg between 2010 and 2015 were included in the present study. All of the patients who underwent a bariatric procedure between 2010 and 2014 were used as training data. Data from patients who underwent a bariatric surgical procedure in 2015 were used as test data to validate the algorithm’s performance in predicting severe postoperative complication within 30 days after surgery. In total, 37,811 and 6250 bariatric patients from SOReg were included in the training data and test data, respectively. In total, 16 features were included in ML, including five continuous features (age, HbA1c, body mass index (BMI), waist circumference (WC), and operation year) and 10 binary features (sleep apnoea, hypertension, diabetes, dyslipidaemia, dyspepsia, depression, musculoskeletal pain, previous venous thromboembolism, revisional surgery, and severe postoperative complication). The last binary feature, i.e., severe postoperative complication, was used as an output variable for the supervised ML classifiers. For training data, the binary features were converted into dummy variables, and the continuous features were standardized to have the mean 0 and standard deviation 1 before they enter the classifier. For test data, the continuous features were standardized using the corresponding means and standardizations from the training data. HbA1c was log transformed before standardization, because of its asymmetric distribution.

### 2.2. Descriptive and Inferential Statistical Methods

The demographic and baseline characteristics of the patients were presented using descriptive statistical methods. Continuous variables were portrayed as mean and standard deviation (SD), or median and interquartile range where suitable, while the categorical variables were outlined as the counts and percentages. The difference between the patient presenting and without severe postoperative complication was tested using the Student’s *t*-test or the Mann-Whitney *U* test for normally or asymmetrically distributed continuous variables, respectively; and, the χ^2^ test was while used for binary variables.

### 2.3. Machine Learning (ML) Algorithms

In the current study, eight base ML algorithms, i.e., logistic regression, linear discriminant analysis (LDA), quadratic discriminant analysis (QDA), decision tree, k-nearest neighbor (KNN), support vector machine (SVM), multilayer perceptron (MLP), and deep learning neural network (NN), and 11 ensemble algorithms, i.e., adaptive boosting (AdaBoost) logistic regression, bagging LDA, bagging QDA, random forest, extremely randomized (Extra) trees, AdaBoost Extra trees, gradient regression tree, AdaBoost Gradient trees, bagging KNN, AdaBoost SVM, and bagging MLP, were implemented [27,28].

### 2.4. Ensemble Learning

In order to improve the generalizability and robustness over a single ML algorithm, we also used ensemble methods to combine multiple base or ensemble algorithms. Five ensemble methods were applied in our study:

1. AdaBoost for logistic regression, Extra trees, gradient regression trees, and SVM [29];
2. bagging for LDA, QDA, KNN, and MLP [30];
3. random forests for decision tree [31];
4. Extra trees for decision tree [32]; and,
5. gradient boosted regression trees for decision tree [33].

### 2.5. Initialization and Optimization of Hyperparameters

ML algorithms involve a number of hyperparameters that have to be fixed before running the algorithms. In contrast to the parameters that are learned by training, hyperparameters determine the structure of a ML algorithm and how the algorithm is trained. The initial values of the hyperparameters for each ML algorithm used in our study are the default values that are specified in the employed software packages based on recommendations or experience [34]. In the KNN algorithm, the ten nearest neighbors were used. In the MLP algorithm, two hidden layer were used with five and two neurons, respectively. In the deep learning NN algorithm, the sequential linear stack of layers was used, with five hidden layers (three dense layers and two dropout regularization layers). For the detailed hyperparameterization of the algorithms, please refer the scikit-learn user manual at http://scikit-learn.org/stable/supervised_learning.html [35] and the Keras Documentation at https://keras.io/.

The hyperparameter optimization is defined as a tuple of hyperparameters that yields an optimal algorithm that minimizes a predefined loss function (i.e., cross entropy loss function in our study, see Appendix A) on a held-out validation set of the training data. However, the most wildly used exhaustive grid search was used to perform hyperparameter optimization in our study, which specified the subset of the hyperparameter space of a ML algorithm and it was evaluated by cross-validation using the training data [36].

### 2.6. Cross-Validation

For the training data, *k*-fold (*k* = 5 in our analysis) cross-validated predictions were used as predicted values. This approach involves randomly dividing the training data into *k* groups, or folds, of approximately equal size. Afterwards, an algorithm is trained on the *k*-1 folds and the rest one fold is retained as the validation fold for testing the algorithm. The process is repeated until the algorithm is validated on all the *k* folds. For each patient in the training data, the predicted value that he/she obtained is the prediction when he/she was in the validation fold. Therefore, only cross-validation strategies that assign all the patients to a validation fold exactly once can be used for the cross-validated prediction [27].

### 2.7. Synthetic Minority Oversampling Technique

The bariatric surgery data is extremely imbalanced, i.e., only 1408 of 44,061 (3.2%) patients experienced severe postoperative complication after bariatric surgery. The imbalance often results in serious bias in the performance metrics [37]. Therefore, we performed the synthetic minority oversampling technique (SMOTE) to tackle the imbalance [38]. SMOTE generates a synthetic instance by interpolating the *m* instances (for a given integer value *m*) of the minority class that lies close enough to each other to achieve the desired ratio between the majority and minority classes. In our study, a 1:1 ratio between the patients presenting severe postoperative complication and without severe postoperative complication was achieved in the training data, i.e., SMOTE training data. The aforementioned nine of the 11 ensemble ML algorithms and the deep learning NN were also implemented for the SMOTE training data.

### 2.8. Performance Metrics

The performances of the 29 ML algorithms were evaluated using accuracy, sensitivity, specificity, and area under the receiver operating characteristic (ROC) curve. ROC curve shows the trade-off that the algorithms set the different threshold values for the posterior probability for the prediction.

Appendix A gives the terminology and derivations of accuracy, sensitivity, specificity, and area under the ROC curve.

### 2.9. Software and Hardware

The descriptive and inferential statistical analyses were performed using Stata 15.1 (StataCorp LLC, College Station, TX, USA). The ML algorithms were achieved using packages scikit-learn 0.19.1 (scikit-learn, http://scikit-learn.org/) [35] and Keras 2.1.6 (Keras, https://keras.io/) in Python 3.6 (Python Software Foundation, https://www.python.org/).

All of the computation was conducted in a computer with 64-bit Windows 7 Enterprise operation system (Service Pack 1), Intel^®^ Core^TM^ i5-4210U CPU @ 2.40 GHz, and 16.0 GB installed random access memory.

The running time of the ML method highly relies on the number of observations and variables, version of software, initialization of hyperparameters, and hardware. In our study, with the default hyperparameters being used by the machine learning packages, the running time ranged from <1 min for logistic regression (without oversampling) to 7 h for deep learning NN (with oversampling) on our computer.

### 2.10. Ethics Approval

The Regional Ethics Committee in Stockholm (approval number: 2013/535-31/5) approved the study and it was conducted in accordance with the ethical standards of the Helsinki Declaration (6th revision).

## 3. Results

Table 1 and Table 2 present the baseline characteristics of the patients in the training data and the test data. The percentages of severe complication in the two data sets are 3.2% and 3.0%, respectively. No statistically significant difference was found for the percentages of severe complication between the two data sets (Pearson chi-square = 0.8283, *p* = 0.363).

Univariable analyses indicate that the differences of mean age, BMI, median HbA1c, percentages of comorbidities for hypertension, diabetes, dyslipidaemia, and previous venous thromboembolism, and percentage of revisional surgery between the patients presenting and without severe complication are statistically significant in the training data (Table 1). In the test data, the statistically significant differences were found for age, WC, HbA1c, dyslipidaemia, and revisional surgery (Table 2).

Multivariable logistic regression analysis for the same data was published elsewhere [6]. In brief, revisional surgery, age, low BMI, operation year, WC, and dyspepsia were associated with an increased risk for severe postoperative complication; however, the performance of the multivariable logistic regression model for predicting the risk in individual patient case was poor. The validation of the model tested on patients operated in 2015 resulted in an area under the ROC curve of only 0.53, a Hosmer–Lemeshow goodness of fit 17.91 (*p* = 0.056) and Nagelkerke R^2^ 0.013 [6].

**Figure 1:**
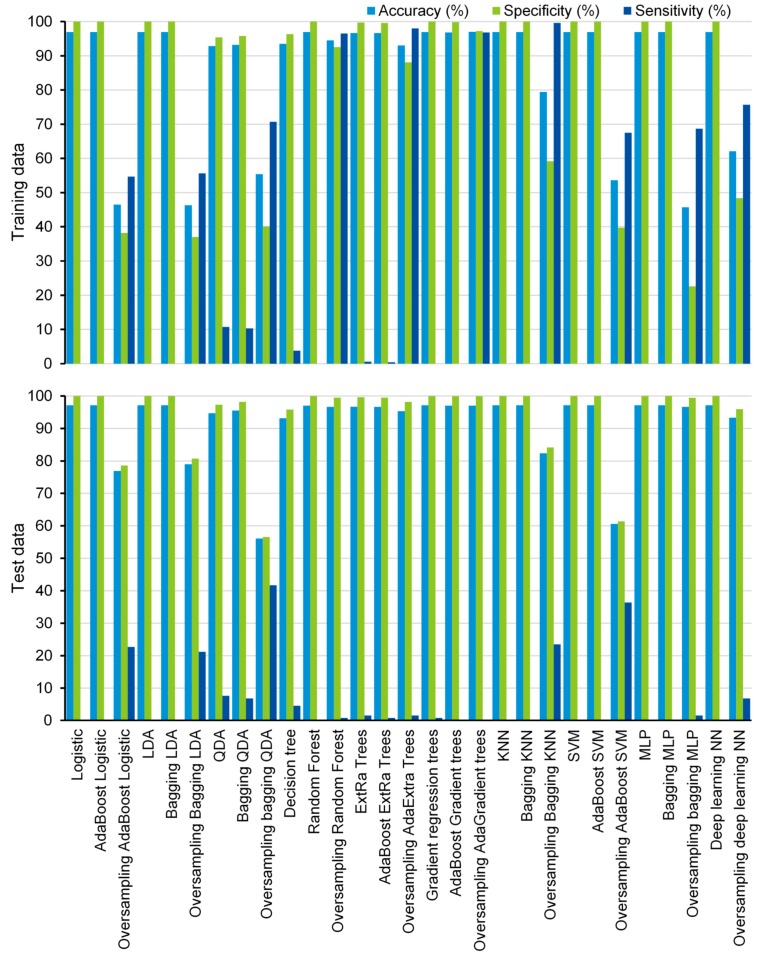
Performance of the algorithms. AdaBoost—adaptive boosting; LDA—linear discriminant analysis; QDA—quadratic discriminant analysis; ExtRa—extremely randomized; AdaExtra—adaptive boosting extremely randomized; AdaGradient—adaptive boosting gradient; KNN—k-nearest neighbor; SVM—support vector machine; MLP—multilayer perceptron; NN—neural network.

In the current study, 19 supervised machine learning algorithms were compared and ten of them were also trained using the SMOTE, resulting in 29 ML algorithms. Most of the machine learning algorithms have shown high accuracy (>90%) and specificity (>90%) for both training data and test data (Figure 1), except that bagging LDA, bagging QDA, AdaBoost SVM, and MLP show low accuracy (<60%) for SMOTE training data, and oversampling-based bagging QDA shows low accuracy for test data (accuracy = 56.1%) (Figure 1).

Although most of the algorithms have shown low sensitivity for both the training data and the test data, some of them exhibited promising prediction ability in the training data. The sensitivities of oversampling-based bagging QDA, random forest, AdaBoost extremely randomized (AdaExtra) trees, AdaBoost gradient regression (AdaGradient) trees, bagging KNN, and deep learning NN are 70.7%, 96.5%, 98.0%, 96.8%, 99.6%, and 75.7% for the SMOTE training data, respectively (Figure 1). Even for test data, oversampling-based bagging QDA and AdaBoost SVM show significantly higher prediction ability than other algorithms. The sensitivities of the two algorithms are 41.7% and 36.4%, respectively (Figure 1). However, they still do not achieve an acceptable level for practical application.

**Figure 2:**
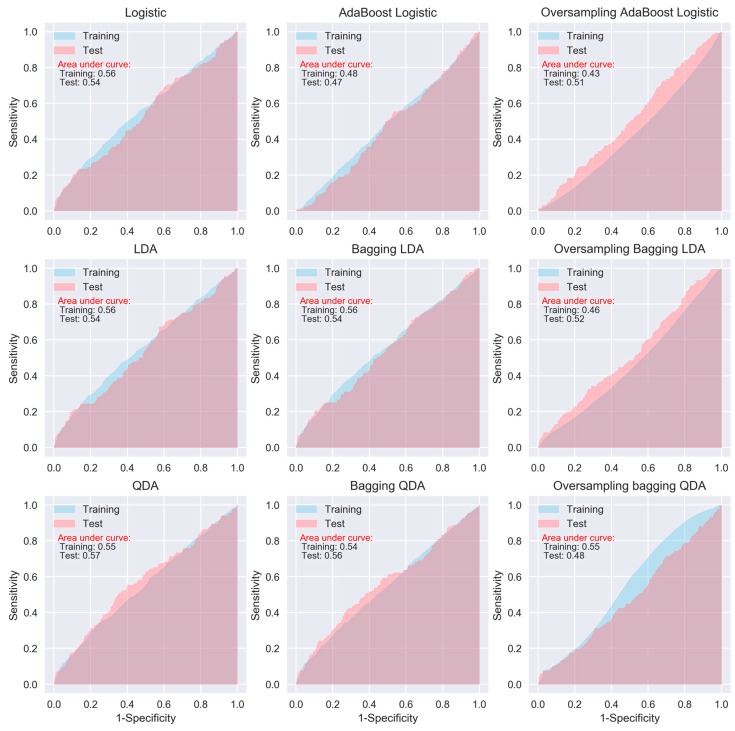
Receiver operating characteristic (ROC) curves of logistic regression, LDA, and QDA.

**Figure 3:**
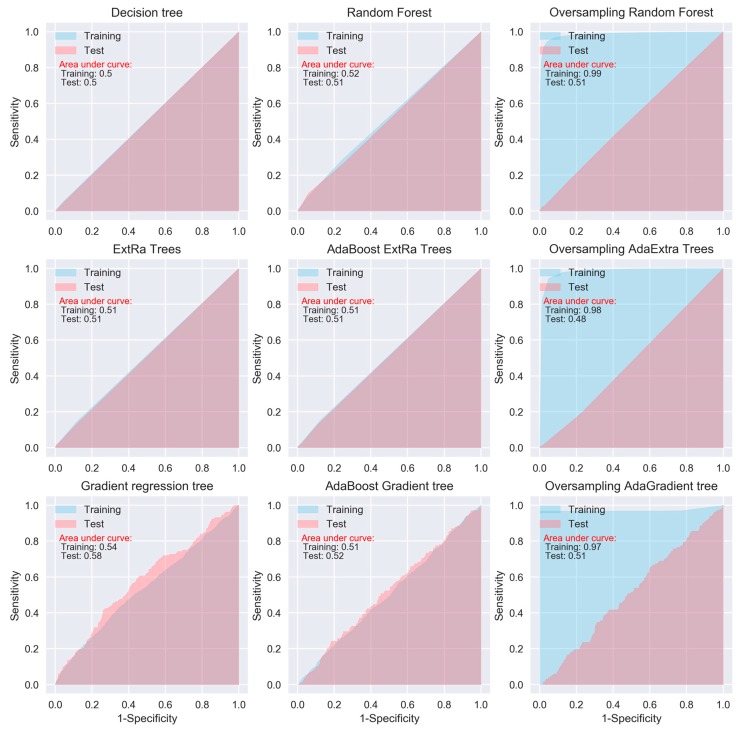
ROC curves of tree-based algorithms.

**Figure 4:**
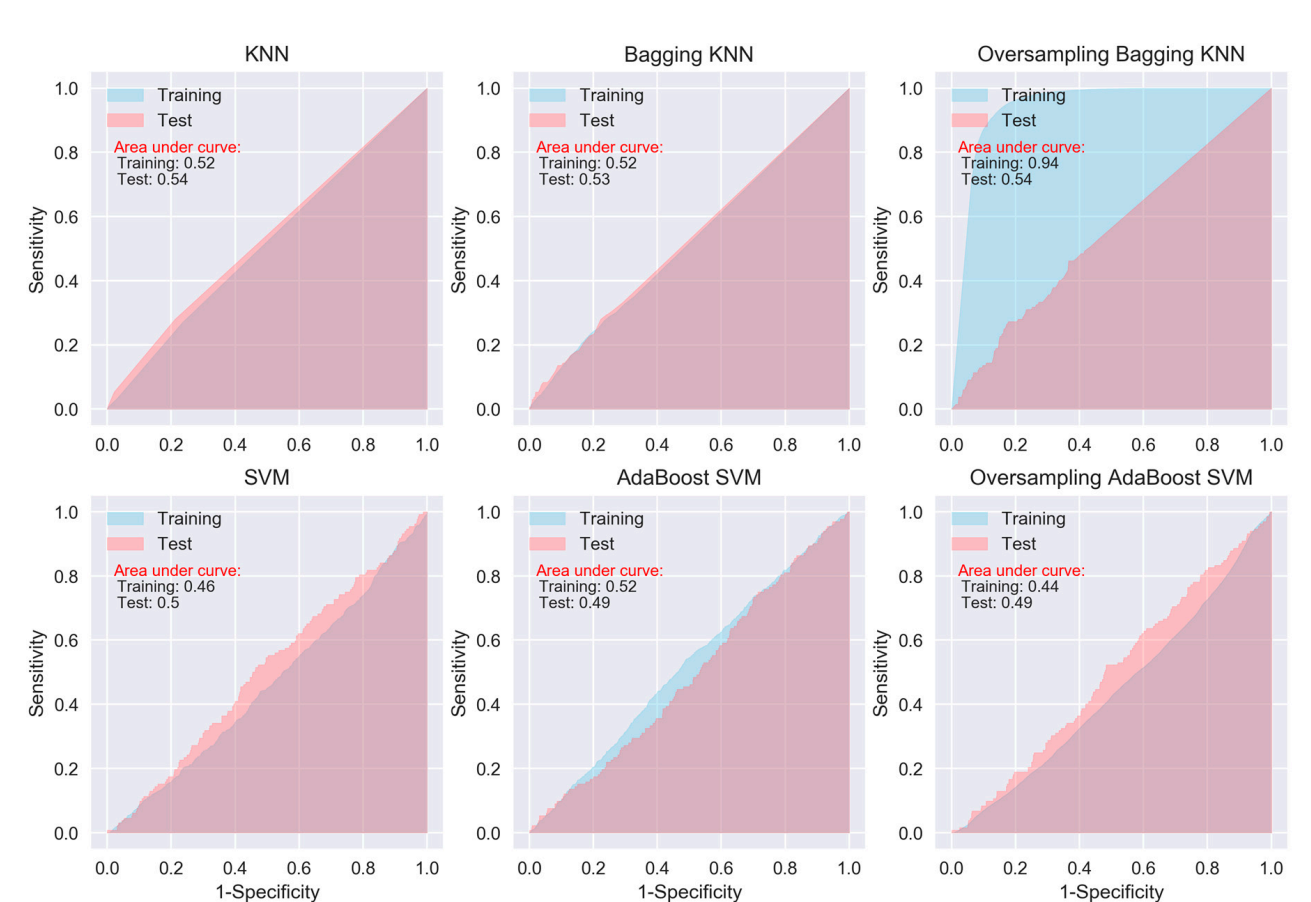
ROC curves of KNN and SVM.

**Figure 5:**
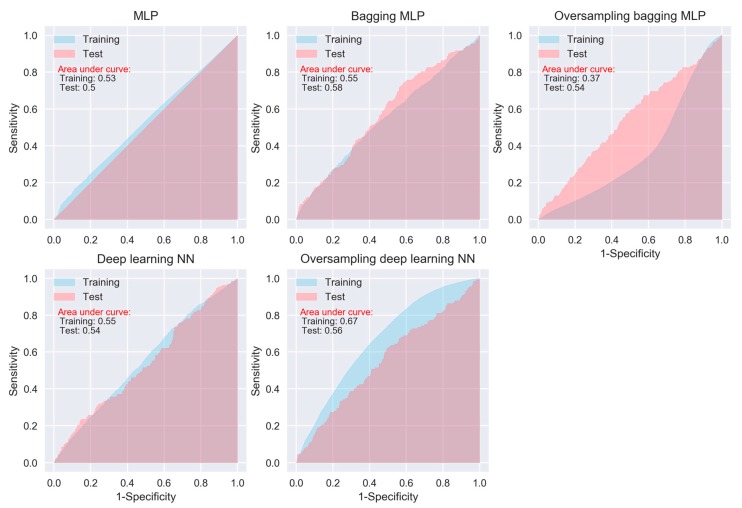
ROC curves of neural network algorithms.

When considering sensitivity and specificity together, most of the algorithms did not show better prediction ability than a random predictor, i.e., an area under ROC curve of 0.5. The areas under the ROC curves for all of the algorithms, except for the oversampling-based random forest, AdaExtra trees, and AdaGradient trees, and KNN, are around 0.5 (Figure 2, Figure 3, Figure 4 and Figure 5). Although the oversampling-based random forest, AdaExtra trees, AdaGradient trees, and KNN show outstanding prediction ability on the SMOTE training data (areas under ROC curves are above 0.9), their performances on the test data are not optimistic (Figure 3 and Figure 4).

The performance of the three regression-based algorithms (logistic regression, LDA, QDA), SVM, and the two neural network-based algorithms (MLP and deep learning NN) was poor in any situation. However, the bagging MLP and deep learning NN outperforms the tree-based algorithms (Figure 3 and Figure 5) for the test data, their areas under ROC curves for the test data are 0.58 and 0.56, respectively (Figure 5), which are greatest among all of the algorithms.

## 4. Discussion

Historically, laparoscopic gastric bypass has, for a long time, been the most common bariatric procedure in Sweden, although laparoscopic sleeve gastrectomy has increased in popularity over more recent years [3,39]. The surgical technique is highly standardized, with more than 99% of all gastric bypass procedures being the antecolic, antegastric, or laparoscopic gastric bypass (so called Lönnroth technique) [40]. Virtually all patients receive pharmacologic prophylaxis for deep venous thrombosis and intraoperative antibiotic prophylaxis [3,26]. Patients who have bariatric surgery are exposed to the risk of having postoperative complications, which may increase the complexity of managing the safety and healthcare costs.

Previous studies on postoperative complications of bariatric surgery have mainly used scoring for identifying patients who are more likely to have complications after surgery. However, these methods are not sensitive enough for clinical application [5,6]. Therefore, the potential of ML tools as clinical decision support in identifying risk factors and predicting the health outcomes is worth investigation on complications that are associated with bariatric surgery. To our knowledge, there is only one study that compared the performance of different ML algorithms in predicting the postoperative complications in the imbalanced bariatric surgery dataset [22]. Although the study indicates that the combination of a suitable feature selection method with the ensemble ML algorithm equipped with SMOTE can achieve higher performance in predictive models for bariatric surgery risks, the ML algorithms were not validated while using external test data. After all, for prediction purpose, we are not very interested in whether or not an algorithm accurately predicts a severe complication for patients that are used to train the algorithm, since we already know which of those patients have severe complications, but are interested in whether the algorithms may accurately predict the future patients based on their clinical measurements.

In total, our study compared 29 ML algorithms using real world data. Although the sensitivities of the algorithms were generally low, the study indicates that some of the ML algorithms were able to achieve higher accuracy than the traditional logistic regression models [5,6,41]. Four of the 29 algorithms were able to achieve high sensitivity (95%) and two achieved moderate sensitivity (>70%) in the training data, including three tree-based algorithms, bagging KNN, bagging QDA, and deep learning NN. We should notice that all of the high or moderate sensitivities were obtained from the SMOTE training data and/or using ensemble algorithms. Our findings support the previous study that ensemble ML algorithms equipped with SMOTE can achieve higher performance metrics for imbalanced data [22]. However, we should also keep in mind that accuracy is a function of the incidence, and hence the same predictor can provide different accuracy when the incidence changes. It might be the reason that some of the algorithms perform worse for SMOTE data.

Despite showing promising capability of prediction in the training data, none of the 29 ML algorithms satisfactorily predicted severe postoperative complication after bariatric surgery in the test data. Why did the algorithms do a poor job of predicting the patients who had severe complication in test data? One potential explanation for this may be related to the limited number of severe postoperative complications in the current dataset, which cannot reveal the underlying relationship between risk factors and adverse health outcomes. Although there are several known risk factors, each of them only imposes a small increase in the risk for postoperative complication [3,4,42,43]. Another likely explanation may be that preoperatively known variables are insufficient in predicting postoperative complications. In previous studies, the highest accuracy for the prediction of postoperative complication has been models including operation data, mainly intraoperative complication and conversion to open surgery [3,5]. Although including intraoperative adverse events and conversion to open surgery may improve the accuracy of the prediction models, such models would not be useful in the preoperative assessment for patients or for case mix comparisons. Furthermore, because the algorithms try to minimize the total error rate out of all classes, irrespective of which class the errors come from, they are not appropriate for imbalanced data, such as what we used in our study [44].

When compared with traditional generalized linear predictive models, non-linear ML algorithms are more flexible and they may attain higher accuracy, but at the expense of less interpretability. Although there are interpretable models, such as regression, Naïve Bayes, decision tree, and random forests, several models are not designed to be interpretable [27]. The aim of the methods is to extract information from the trained model to justify their prediction outcome, without knowing how the model works in detail. The trade-off between prediction accuracy and model interpretability is always an issue when we have to consider in building a ML algorithm. A common quote on model interpretability is that, with an increase in model complexity, model interpretability goes down at least as fast. Fully nonlinear methods, such as bagging, boosting, and support vector machines with nonlinear kernels are highly flexible approaches that are harder to interpret. Deep learning algorithms are notorious for their un-interpretability due to the sheer number of parameters and the complex approach to extracting and combining features. Feature importance is a basic (and often free) approach to interpreting the model. Although some nonlinear algorithms, such as tree-based algorithms (e.g., random forest), may allow for obtaining information on the feature importance, we cannot obtain such information from many ML algorithms.

Therefore, recent attempts have been made to improve the interpretability for the black-box algorithms, even including deep learning. Local interpretable model-agnostic explanations (LIME) is one of them to make these complex models at least partly understandable. LIME is a more general framework that aims to make the predictions of ‘any’ ML model more interpretable. In order to remain model-independent, LIME works by locally modifying the input to the model [45,46]. Accordingly, instead of trying to understand the entire model at the same time, a specific input instance is modified and the impact on the predictions are monitored.

Regarding specific algorithm, although their motivations differ, the logistic regression and LDA or QDA methods are closely connected; therefore, we were not surprised that LDA or QDA did not show significant improvement in prediction than logistic regression [6]. KNN takes a complete different approach from classification, which is completely non-parametric [27]. Therefore, we can expect it to outperform parametric models, such as logistic and LDA. However, KNN cannot tell us which predictor are of importance. QDA serves as a compromise between the non-parametric KNN and the LDA and logistic regression. Though not as flexible as KNN, QDA can perform better in the limited training data situation. MLP is a class of feedforward artificial neural network, which consists of at least three layers of nodes. Its multiple layers and non-linear activation can distinguish data that is not linearly separable. Deep learning NNs are high-level NNs, which include convolutional NN and recurrent NN et al. In our study, the deep learning NN with five hidden layers outperforms the conventional MLP with two hidden layers, especially on SMOTE training data (areas under ROC curves are 0.67 vs. 0.37), which deserves further investigation in the future.

Our study demonstrates that ensemble learning may improve predictions by combining several base algorithms. However, usually there are several ensemble methods available, such as bagging, boosting, and stacking [47]. A number of studies have shown that, AdaBoost is more effective at reducing bias, bagging is more effective at reducing variance, and stacking may improve predictions, in general, when decomposing a classifier’s error into bias and variance terms [30]. There is no golden rule on which method works best. The choice of specific ensemble methods is case by case and depends enormously on the data.

There are some limitations in our study, and we would like to highlight them below:

First, the explanatory variables that were used to predict the occurrence of severe complications have shown a very low predictive ability in terms of the Nagelkerke R^2^ and ROC curve measure in our previous study [6]. With such a low predictive ability to start with, we cannot expect a dramatic improvement in sensitivity while using currently available data.

Second, the event rate in our study is very low, i.e., only 3% of patients experienced severe complications. This means that, if we simply predict that all patients will have no severe complication, we would have 97% accuracy and 100% specificity. Therefore, how to improve sensitivity of the algorithms is the most important goal in our future study.

Third, the study was limited to data registered within the SOReg. Cardiovascular and pulmonary comorbidities other than sleep apnea are not mandatory variables within the registry, and they could thus not be included in the model. Although these comorbidities are known risk factors for postoperative complications [4,43,48], they are not highly prevalent in European studies [5]. Given the low predictive ability of the currently available variables, including other background data, such as birth country, education level, income, and previous diseases from other Swedish registries that would have increased the predictive ability of the algorithms.

Fourth, although we compared 29 ML algorithms that were investigated in our study, they are convenient and feasible methods for general medical researchers. Due to computational complexity and less interpretability, many complicated and advanced ML algorithms were not investigated in our study. However, our study at least points out a promising way for future investigations, i.e., deep NN equipped with SMOTE.

Fifth, the exhaustive grid search was used in our hyperparameter optimization, which is extremely resource consuming and not optimal for complex ML algorithms; therefore, other advanced methods, such as gradient-based or evolutionary optimization, may be considered in the future.

Furthermore, because choosing a feature scaling method completely depends on the numerous aspects of the data, it is also worthy to compare the currently used standardization in our study with other scaling methods, such as mean normalization, min-max scaling, or unit vector, in future studies.

## 5. Conclusions

When compared to other ML algorithms, deep NN has the potential to improve the accuracy in predicting the severe postoperative complication among bariatric surgery patients. The ensemble algorithms outperform base algorithms. Due to the imbalanced nature of the data where the number of the interested outcome is relatively small, the oversampling technique needs to be adopted to balance the dichotomous outcomes.

## Figures and Tables

**Table 1:** Base line characteristics of the training patients.

Characteristics	All N = 37,811	No Serious Complication N = 36,591 (96.8%)	Having Serious Complication N = 1220 (3.2%)	*p*-Value
Age in years, mean ± SD	41.2 ± 11.2	41.1 ± 11.2	42.9 ± 10.7	<0.001 *
Sex, *n* (%)				
Female	28,682 (75.9%)	27,766 (75.9%)	916 (75.1%)	0.521 ^†^
Male	9129 (24.1%)	8825 (24.1%)	304 (24.9%)	
BMI in kg/m^2^, mean ± SD	42.12 ± 5.66	42.13 ± 5.66	41.79 ± 5.58	0.0355 *
WC in cm, mean ± SD	126.0 ± 14.0	126.0 ± 14.0	126.2 ± 13.8	0.6018 *
HbA1c, median (P25, P75)	38 (35, 42)	38 (38, 32)	38 (35, 43)	0.0090 ^‡^
Comorbidity, *n* (%)				
Sleep apnoea	3792 (10.0%)	3656 (10.0%)	136 (11.2%)	0.186 ^†^
Hypertension	9760 (25.8%)	9404 (25.7%)	356 (29.2%)	0.006 ^†^
Diabetes	5407 (14.3%)	5204 (14.2%)	203 (16.6%)	0.018 ^†^
Dyslipidaemia	3802 (10.1%)	3667 (10.0%)	135(11.1%)	0.233 ^†^
Dyspepsia	3970 (10.5%)	3803 (10.4%)	167 (13.7%)	<0.001 ^†^
Depression	5609 (14.8%)	5409 (14.8%)	200 (16.4%)	0.119 ^†^
Musculoskeletal pain	4905 (13.0%)	4754 (13.0%)	151 (12.4%)	0.529 ^†^
Previous venous thromboembolism	918 (2.4%)	875 (2.39%)	43 (3.52%)	0.011 ^†^
Revisional surgery	1367 (3.6%)	1261 (3.5%)	106 (8.7%)	<0.001 ^†^

SD—standard deviation; BMI—body mass index; WC—waist circumference; P25—the 25th percentile; P75—the 75th percentile. * *t*-test was used; ^†^ χ^2^ test was used; ^‡^ Mann-Whitney *U* test was used.

**Table 2:** Base line characteristics of the test patients.

Characteristics	All N = 6250	No Serious Complication N = 6062 (97.0%)	Having Serious Complication N = 188 (3.0%)	*p*-Value
Age in years, mean ± SD	41.2 ± 11.5	41.2 ± 11.5	42.9 ± 11.8	0.0423 *
Sex, *n* (%)				
Female	4832 (77.3%)	4682 (77.2%)	150 (79.8%)	0.411 ^†^
Male	1418 (22.7%)	1380 (22.8%)	38 (20.2%)	
BMI in kg/m^2^, mean ± SD	41.22 ± 5.87	41.20 ± 5.89	41.95 ± 5.40	0.0848 *
WC in cm, mean ± SD	123.3 ± 14.1	123.2 ± 14.0	126.2 ± 14.7	0.0086 *
HbA1c, median (P25, P75)	37 (34, 41)	37 (34, 41)	38 (35, 44)	0.0017 ^‡^
Comorbidity, *n* (%)				
Sleep apnoea	622 (10.0%)	607 (10.0%)	15 (8.0%)	0.359 ^†^
Hypertension	1563 (25.0%)	1506 (24.8%)	57 (30.3%)	0.088 ^†^
Diabetes	761 (12.2%)	734 (12.1%)	27 (14.4%)	0.352 ^†^
Dyslipidaemia	518 (8.3%)	493 (8.13%)	25 (13.3%)	0.011 ^†^
Dyspepsia	645 (10.3%)	620 (10.2%)	25 (13.3%)	0.173 ^†^
Depression	1096 (17.5%)	1053 (17.4%)	43 (22.9%)	0.051 ^†^
Musculoskeletal pain	1315 (21.0%)	1268 (20.9%)	47 (25.0%)	0.176 ^†^
Previous venous thromboembolism	182 (2.9%)	177 (2.99%)	5 (2.7%)	0.834 ^†^
Revisional surgery	61 (1.0%)	54 (0.9%)	7 (3.7%)	<0.001 ^†^

SD—standard deviation; BMI—body mass index; WC—waist circumference; P25—the 25th percentile; P75—the 75th percentile. * *t*-test was used; ^†^ χ^2^ test was used; ^‡^ Mann-Whitney *U* test was used.

## Data Availability

Aggregated numerical data are presented in Table 1 and Table 2. The ethical approval obtained for this study prevents the human data being shared publicly to protect patients’ privacy. Interested readers can contact Dr. Erik Stenberg with their research plan to request access. This would be passed to the ethics committee who will decide whether they can access the data directly from the relevant Swedish authority.

## Appendix A

### Terminology and Derivations

Cross Entropy loss (Log loss):

(A1) $$V\left(f\left(\vec{x}\right),y\right)=-y\ln\left(f\left(\vec{x}\right)\right)-\left(1-y\right)\ln\left(1-f\left(\vec{x}\right)\right)$$

where *y* is true classifier ∈{0,1} and $f\left(\vec{x}\right)$ is predicted value.

True or false refers to the predicted outcome being correct or wrong, while positive or negative refers to presenting severe complication or no severe complication.

(A2) $$\mathrm{ACC}=\frac{\mathrm{TP}+\mathrm{TN}}{\mathrm{Total}}\times 100\%$$

(A3) $$\mathrm{SEN}=\frac{\mathrm{PT}}{P}$$

(A4) $$\mathrm{SPE}=\frac{\mathrm{TN}}{N}$$

(A5) $$\mathrm{AUC}=\int_{0}^{1}\mathrm{SE}N_{T}{\left(1-\mathrm{SPE}\right)}_{T}\mathrm{dT}$$

ACC	accuracy
SEN	sensitivity
SPE	specificity
TP	number of true positives, i.e., patient presenting severe complication correctly predicted as positive
TN	number of true negatives, i.e., patient without severe complication correctly predicted as negative
FP	number of false positives, i.e., patient without severe complication wrongly predicted as positive
FN	number of false negatives, i.e., patient presenting severe complication wrongly predicted as negative
Total	total number of the patients, i.e., TP + TN + FP + FN
P	number of patients presenting severe complication, i.e., TP + FN
N	number of patients without severe complication, i.e., TN + FP
AUC	area under the receiver operating characteristic (ROC) curve for binary outcome
T	threshold for a patient is classified as presenting severe complication if X > T, where X is predicted probability of a patients presenting severe complication by an algorithm.
